# Editorial: World antimicrobial awareness week

**DOI:** 10.3389/fpubh.2024.1543642

**Published:** 2025-01-07

**Authors:** Dalal Hammoudi Halat, Issmat I. Kassem, Marwan Osman, Vera Manageiro

**Affiliations:** ^1^Academic Quality Department, QU Health, Qatar University, Doha, Qatar; ^2^Department of Food Science and Technology, Center for Food Safety, University of Georgia, Griffin, GA, United States; ^3^Department of Neurosurgery, Yale University School of Medicine, New Haven, CT, United States; ^4^National Reference Laboratory of Antibiotic Resistances and Healthcare-Associated Infections, Department of Infectious Diseases, National Institute of Health Doutor Ricardo Jorge, Lisbon, Portugal; ^5^Centre for the Studies of Animal Science, Institute of Agrarian and Agri-Food Sciences and Technologies, University of Porto, Porto, Portugal; ^6^AL4AnimalS, Associate Laboratory for Animal and Veterinary Sciences, Lisbon, Portugal

**Keywords:** antimicrobials, antimicrobial resistance, One Health, surveillance, awareness, epidemiology

## 1 Introduction

The World Antimicrobial Awareness Week (WAAW), established at the 68th World Health Assembly in 2015 and observed from the 18th to the 24th of November each year, has been an annual occasion to direct public attention to the overwhelming burden of antimicrobial resistance (AMR). The key aim of this event is to improve awareness to AMR, highlighting the importance of education and communication. In 2023, the Food and Agriculture Organization of the United Nations (FAO), the United Nations Environment Program (UNEP), the World Health Organization (WHO), and the World Organization for Animal Health (WOAH) announced the rebranding of WAAW as the World AMR Awareness Week. These changes aimed at better supporting the challenges of AMR (1). A breakthrough in bacterial AMR epidemiology were the comprehensive analyses of the global burden through 2050 (2). These studies highlighted a concerning increase in resistance to critically important antimicrobials, particularly carbapenem-resistant Gram-negative bacteria and multidrug-resistant *Mycobacterium tuberculosis*. Alarmingly, bacterial infections were estimated to contribute to ~5 million deaths annually, with ~1.2 million directly attributed to AMR (2). Efforts to prevent AMR must remain a top priority for global health stakeholders, because AMR represents one of the top critical challenges of the 21st century, particularly in low-resource settings and conflict-affected regions (3). Strengthening surveillance systems is essential to track resistance patterns, identify emerging threats, and guide evidence-based targeted interventions. Equally important is fostering awareness among healthcare providers, policymakers, and the broader community about the risks posed by AMR and the urgent need for responsible antimicrobial use. These measures, alongside robust antimicrobial stewardship programs, are essential to mitigating the growing impact of AMR, reducing associated morbidity and mortality, and safeguarding global health.

This Research Topic aligns with WAAW's mission, showcasing multidisciplinary studies that emphasize awareness, innovation, and collaboration. The articles within this Research Topic highlight critical interventions, insights into surveillance systems, and the socio-cultural dimensions of AMR, providing a holistic perspective to inform and inspire action.

## 2 Key contributions to understanding and addressing AMR

### 2.1 Policy and behavioral interventions

Effective AMR management relies on robust policies and public engagement. Different perspectives of AMR are addressed in this Research Topic, reflecting broad research venues that are relevant to a broad scientific readership. For example, a comparison of factors that contribute to the success of AMR interventions among high and low-middle income countries is presented by Graells et al.. In these countries, entrepreneurs have an instrumental role to play in curbing AMR through building of political will, and nourishing enthusiasm and drive for the required momentum of AMR policy adoption as argued by Otaigbe. Experience with new diagnostics to support community management of respiratory tract infections, and thereby emerging resistance, is discussed in a qualitative assay by Hoste et al.. Tenzin et al. use knowledge, attitudes, and practice research to investigate how competent adults perceive the use of antimicrobial agents and AMR, focusing on community pharmacy role in meeting national plans to mitigate AMR. Likewise, a similar screening of public opinions toward AMR was conducted by Singh-Phulgenda et al., culminating in the recommendation to organize targeted awareness campaigns and educational initiatives that address AMR knowledge gaps and promote responsible antibiotic use, highlighting the key role of the general population in combating AMR.

### 2.2 Surveillance and data-driven insights

Across the world, countries have been encouraged to develop national plans to mitigate AMR (4). In this regard, surveillance systems have been considered vital to monitor AMR trends, evaluate effects of various interventions, and inform decision making by stakeholders (5). In this Research Topic, the United Arab Emirates (UAE) AMR Surveillance Consortium's contributions demonstrated how integrated surveillance systems can track resistance in pathogens like methicillin-resistant *Staphylococcus aureus*, *Streptococcus pneumoniae*, vancomycin-resistant enterococci, *Acinetobacter* species, *Pseudomonas aeruginosa*, carbapenem-resistant *Enterobacterales* as well as *Mycobacterium tuberculosis*. Indeed, Thomsen et al. described a broad network of over 300 surveillance centers. Furthermore, the Consortium presented trends of resistance in the UAE over 12 years for seven different bacterial pathogens, leveraging a unified application for the management and analysis of microbiology laboratory data with a particular focus on AMR surveillance. This application was developed and supported by the WHO Collaborating Center for Surveillance of AMR. The investigations called for further epidemiological enquiry and monitoring of genetic evolution in these pathogens to provide new strategies for prevention and control. Also, a retrospective 3-year study of highly resistant *Candida auris* in the UAE is presented, calling for enhanced infection control measures to prevent continued dissemination of this urgent threat pathogen that is characterized by high mortality and persistent transmissions in healthcare settings. The rising incidence and burden of fungal infections was also highlighted by Husni et al. in a multicenter study from Lebanon, uncovering significant increase in non-albicans *Candida* species with high resistance profiles, amidst lack of local treatment guidelines. Consequently, the researchers called for establishment of guidelines to decrease morbidity and mortality as well as for continuous collection of surveillance data. Also within the debilitated healthcare system in Lebanon, Daaboul et al. described broad dissemination of *bla*_NDM − 5_ and *bla*_OXA − 244_ genes among carbapenem-resistant *Enterobacterales* in the Lebanese clinical settings, underscoring an urgent necessity for transformative methods to combat AMR in both community and hospital environments.

### 2.3 Innovative therapeutics and risk modeling

The pursuit of novel antimicrobial alternatives is critical considering the limited antimicrobial pipelines. Carbapenem-resistant Gram-negative bacteria are especially worrisome in intensive care units (ICU), as accurately revised by Li et al., making it crucial to increase vigilance against these pathogens during ICU stay, administer antimicrobials rationally based on the pathogen type and susceptibility, and identify carbapenemase types to prevent and control associated infections. Furthermore, a multicenter, retrospective observational study from China by Xiao et al. described bloodstream infections associated with *P. aeruginosa*, showing increased trends in AMR and higher healthcare costs. A prediction model proposed by Sun et al. identified age, hypoproteinemia, daily dose, medication within 14 days prior to surgical intervention, and microbial clearance as significant risk factors for failure of tigecycline therapy of ventilator-associated pneumonia caused by carbapenem-resistant *A. baumannii*. All these studies emphasized the importance of targeted, evidence-based treatment strategies to improve patient outcomes while curbing drug resistance.

### 2.4 One Health perspectives on AMR

AMR nowadays cannot be confronted without considering the interconnectedness between human, animal, and environmental dimensions, often collectively referred to as the One Health approach. This is the collaborative effort of multiple health science professionals to attain optimal health for humans, animals, wildlife, plants, and the environment (6). Such interconnected domains, if abused, contribute to the emergence, evolution, and dissemination of antibiotic-resistant microorganisms on both local and global scale, posing a significant risk factor for global health (7). In this regard, Habiba et al. reported significant use of colistin in poultry farms in Pakistan, leading to rapid shedding through poultry waste to the environment, ultimately affecting biosecurity. This was accompanied by lack of farmers training and experience with antibiotic use and AMR. In Italy, *Salmonella* was identified by Petrin et al. in human, animal, and food samples, with numerous AMR genes and plasmid replicons associated with resistance to critical antimicrobials, favoring their successful spread and complicating the problem of AMR further. Taken together, these studies highlighted the transboundary nature of AMR and the necessity of harmonized surveillance across niches, including food and agriculture.

### 2.5 Cultural and educational strategies

Raising public awareness and addressing cultural issues in mitigating AMR should not be overlooked. In this regard, Waswa et al. described a brief for policymakers about school curricula that advocate for and support integration of AMR content in primary and secondary level grades. The policy brief supports and facilitates efforts by national AMR committees to create more awareness on this issue. Moreover, to highlight tackling AMR in different cultures, Lescure et al. evaluated a communication intervention for general practitioners in multicultural Dutch cities to improve antibiotic prescribing for respiratory tract infections. The intervention proved to be useful and resulted in improvement of self-rated knowledge and learning culturally-sensitive communication skills beneficial to AMR control. As described by Ayorinde et al. in their systematic review on healthcare professionals interactions with patients to discuss AMR, different barriers and facilitators play a role in affecting antimicrobial-associated behaviors by patients, which, if identified and properly addressed, would allow improvement. Taken together, these studies add to the body of evidence investigating and reporting current AMR trends and knowledge and will support ongoing research efforts toward this crucial global health threat.

## 3 Conclusions and a collaborative path forward

In conclusion, by bridging scientific inquiry with advocacy, this Research Topic contributes to the global effort to address AMR. The findings reflect current challenges as well as the immense potential for progress through shared knowledge and concerted action. The articles in this Research Topic highlight the multifaceted nature of AMR and feature the pivotal role of WAAW in sharing evidence, raising awareness, promoting stewardship, and fostering collaborative efforts to combat AMR. While progress has been made, the road ahead requires a stronger emphasis on innovation and technology-driven solutions to secure sustainable outcomes. In this regard, emerging therapeutic strategies, such as the development of novel antibiotics and alternatives to traditional antimicrobials, must be prioritized. Moreover, machine learning and artificial intelligence offer transformative potential in this domain, enabling rapid identification of new antimicrobial compounds and alternative therapeutics needed to enrich the drug pipeline in the face of increased AMR. Advancements in vaccine development also represent a critical preventative measure, reducing reliance on antibiotics and curbing the spread of resistant infections. Within the framework of WAAW, integrating these scientific advances with public health advocacy is essential. Strengthening the alignment between WAAW objectives and cutting-edge scientific developments will reinforce the global fight against AMR, nurturing a more resilient future for global health systems.

## References

[1] World Health Organization. World Antimicrobial Awareness Week (WAAW) Will Now be World AMR Awareness Week. (2023). Available at: https://www.who.int/news/item/06-06-2023-world-antimicrobial-awareness-week-(waaw)-will-now-be-world-amr-awareness-week (accessed November10, 2024).

[2] GBD 2021 Antimicrobial Resistance Collaborators. Global burden of bacterial antimicrobial resistance 1990-2021: a systematic analysis with forecasts to 2050. Lancet. (2024) 404:1199–226. 10.1016/S0140-6736(24)01867-1 39299261 PMC11718157

[3] Osman M Cummings KJ El Omari K Kassem II. Catch-22: war, refugees, COVID-19, and the scourge of antimicrobial resistance. Front Med. (2022) 9:921921. 10.3389/fmed.2022.921921 35814789 PMC9263824

[4] Willemsen A Reid S Assefa Y. A review of national action plans on antimicrobial resistance: strengths and weaknesses. Antimicrob Resist Infect Control. (2022) 11:90. 10.1186/s13756-022-01130-x 35739564 PMC9229779

[5] Al-Haboubi M Glover RE Eastmure E Petticrew M Black N Mays N. Quality and utility of information captured by surveillance systems relevant to antimicrobial resistance (AMR): a systematic review. Antibiotics. (2021) 10:431. 10.3390/antibiotics10040431 33924412 PMC8069834

[6] McEwen SA Collignon PJ. Antimicrobial resistance: a one health perspective. Microbiol Spectr. (2018) 6:10.1128/microbiolspec.arba-0009-2017. 10.1128/microbiolspec.ARBA-0009-2017 29600770 PMC11633550

[7] Aslam B Khurshid M Arshad MI Muzammil S Rasool M Yasmeen N. Antibiotic resistance: One Health one world outlook. Front Cell Infect Microbiol. (2021) 11:771510. 10.3389/fcimb.2021.771510 34900756 PMC8656695

